# Global burden of hearing loss in people aged 60 years and older, 1990–2021: results from the global burden of disease study

**DOI:** 10.3389/fpubh.2025.1606673

**Published:** 2025-11-26

**Authors:** Zhi-qiang Zhang, Jing-yang Li, Hongyou Wang, Chi-yi Fu, Ya-Lun Li, Qian Guo, You-wei Bao, Jun Wu, Jun-chao Liao, Yu-qi Song, Dong-xu Li, Xin-hua Zhu

**Affiliations:** 1 Department of Otorhinolaryngology, Head and Neck Surgery, The Second Affiliated Hospital, Jiangxi Medical College, Nanchang University, Nanchang, China; 2 Department of Clinical Medicine, The First Clinical Medical College, Nanchang University, Nanchang, China; 3 Department of Anesthesiology, The First Hospital of Jilin University, Changchu, China; 4 Southwest Medical University, Luzhou, China; 5 Department of Rhinology, The First Affiliated Hospital of Zhengzhou University, Zhengzhou, China; 6 Department of Gastrointestinal Surgery, Union Hospital, Tongji Medical College, Huazhong University of Science and Technology, Wuhan, China; 7 Department of Orthopedics, Union Hospital, Tongji Medical College, Huazhong University of Science and Technology, Wuhan, China

**Keywords:** GDB, older adults, hearing loss, HL, global burden of disease (GBD 2021)

## Abstract

**Background:**

Hearing loss (HL) is a significant public health concern, particularly among older adults, where it ranks as the third most common cause of years lived with disability (YLD) globally. This study aims to comprehensively analyze the burden of HL among individuals aged 60 years and older from 1990 to 2021, utilizing data from the Global Burden of Disease (GBD) database.

**Methods:**

We employed a variety of analytical approaches, including descriptive analysis, Joinpoint regression, age-period-cohort analysis, decomposition analysis, and predictive modeling. The study examined trends in incidence, prevalence, and disability-adjusted life years (DALYs) across different socio-demographic index (SDI) quintiles, regions, and genders.

**Results:**

Our findings reveal significant increases in age-standardized rates (ASR) of HL and YLDs over the study period (EAPC = 0.13 and 0.14, respectively), with notable disparities across SDI categories. The burden of HL was highest in low SDI countries, where socio-economic factors severely impacted access to hearing care. Gender differences were pronounced, with males exhibiting higher prevalence rates than females. Predictive analysis indicates a continued rise in HL cases and associated YLDs (projected to reach 46.9 million YLDs by 2040), emphasizing the urgent need for targeted public health interventions.

**Conclusion:**

The increasing burden of HL among older adults necessitates enhanced awareness, early detection, and improved access to hearing care services, particularly in low and middle-income countries. Addressing these disparities is crucial for mitigating the socio-economic impacts of HL and improving the quality of life for affected individuals.

## 1 Introduction

Hearing loss is the most common sensory impairment worldwide, affecting an estimated 1.57 billion people in 2019, or one-fifth of the global population. The majority of people with moderate to severe hearing loss are concentrated in the Western Pacific region, along with countries with lower healthcare quality and access (1). Previous studies have shown that hearing loss affects nearly 10% of the adult population (2), that hearing loss is a common health condition in older adults (3), and that senile deafness is the most common type in adults (4). In addition, the prevalence of hearing loss increases significantly with age (2). Hearing loss limits patients in many aspects of daily life and can lead to social exclusion (5). If left unaddressed or unsupported in the context of the individual’s communication needs, it may affect multiple aspects of the individual’s life (1). It has been shown that hearing loss can lead to the development of anxiety and depression (6), especially in older adults (7, 8). Hearing loss also limits opportunities for oral communication and decreases quality of life, raising the risk of dementia and cognitive decline in older adults (9, 10).

However, despite hearing loss being a major global public health problem that requires urgent attention and solutions, there is a lack of a consistent view of epidemiologic trends in hearing loss, especially among older adults (people over 60 years of age). Existing studies are either based on outdated data (11, 12) or limited to specific countries or regions (5), and they lack a scientific, systematic approach to comparing trends in disease burden across different areas. In addition, most studies have aimed to analyze hearing loss in the general population or in children and young adults, whereas few have focused specifically on older adults (13–15). In contrast, our research uses more up-to-date data and more scientifically rigorous statistical methods, covering regions worldwide to comprehensively explore trends in the burden of HL diseases in older adults. This increases the difficulty of developing targeted prevention strategies for different populations and increases the economic burden on society. Therefore, it is particularly important to address differences in scientific research and public health policies to develop targeted prevention strategies for specific populations.

The Global Burden of Disease (GBD) study is a public health program with global reach dedicated to comprehensively assessing and analyzing the burden of disease and injuries worldwide. The latest GBD2021 provides comprehensive, up-to-date epidemiologic data on the burden of 371 diseases and injuries in 204 countries and territories. This study aimed to use the latest data from the GBD2021 project to investigate the burden of hearing loss among people aged 60 years and older globally, regionally, and nationally; characterize trends over time; and project future changes. We consider a variety of factors, including demographic characteristics, socio-economic determinants, and environmental influences, to ensure that the actual burden of disease is accurately reflected.

## 2 Materials and methods

### 2.1 Data sources

All data used in this study were obtained from the Global Burden of Disease (GBD) 2021 database produced by the Institute for Health Metrics and Evaluation (IHME),^1^ which details the data, statistical modeling, and methodology used for inclusion in the study. The GBD database, based on age and gender, includes 204 countries. The GBD database contains 371 diseases and injuries from 204 countries and territories, quantifying and estimating health losses from 1990 to the present by age and sex. The data are standardized to multiple correlates for each disease or injury separately. Censuses, household surveys, civil registration and vital statistics, disease registries, healthcare utilization, air pollution monitoring, satellite imagery, and disease notification from 86,249 sources were used as data sources (16). In addition, we used the Query Exchange tool to obtain data on the incidence, prevalence, and disability-adjusted life years (DALYs) of hearing loss from the GBD database between 1990 and 2021. DisMod-MR is a Bayesian regression algorithm used to assess overall incidence, prevalence, and other metrics in the GBD database in 2021 (17). Specifically, a dataset on hearing loss among people aged 60 years and older from 1990 to 2021 was obtained from the GBD 2021 database.

GBD 2021 uses standardized tools to model processed data for most diseases and injuries, disaggregated by age, sex, location, and year of study. It provides a range of interrelated indicators to assess the burden of disease, including incidence, prevalence, mortality, and years of life lost (YLLs), as well as composite health indicators such as years of life lived with disability (YLDs) and disability-adjusted life years (DALYs). DALYs are widely used as a composite measure of health status and are a useful tool for comparing health disparities across health statuses and populations. DALYs are the total number of years lost due to illness, disability, or premature death and are calculated as the sum of DALYs and DALYs (18).

The SDI we use is a precise indicator that serves as a composite indicator and a composite index that captures national social development from the geometric mean of the following: the total fertility rate for those under 25 years of age, the lagged distribution of per capita income, and the average years of schooling for the population 15 years of age and older, which is then materialized into individual scores on a scale of 0 to 1.1 We use the SDI as an indicator of social development in the countries and regions we include. We artificially categorized the included countries and regions according to five SDI thresholds: high SDI (>0.81), medium-high SDI (0.70–0.81), medium SDI (0.61–0.69), medium-low SDI (0.46–0.60), and low SDI (<0.46), and estimated all the values according to the uncertainty intervals (UIs), which are the values that we The 2nd-5th percentile and 97th-5th percentile of the sample determined the 95% uncertainty intervals of the estimates to derive the distribution values (19). All analyses were conducted using R (version 4.2.2) and adhered to the Guidelines for Accurate and Transparent Health Estimates Reporting (GATHER) to ensure scientific rigor and methodological transparency. According to the established inclusion and exclusion criteria (Supplementary file 1), data on hearing loss among individuals aged 60 years or older were extracted from the GBD database. The detailed data extraction process is shown in Supplementary Figure 1.

### 2.2 Descriptive analysis

We used descriptive analyses to explore spatial and temporal trends in disease and comprehensively analyze and compare the burden of HL across different dimensions, including global HL incidence from 1990 to 2021. Both metrics were assessed by the number of cases, age-standardized or age-adjusted rates (ASRs), and estimated annual percentage change (EAPC) for males, females, and all genders between 1990 and 2021. The Estimated Annual Percentage Change (EAPC) indicates trends in burden at different scales over a given period of time and is a practical way to quantify overall and localized trends in epidemiologic outcomes. In addition, we compared case counts, ASRs, and EAPCs on HL incidence, prevalence, and disability-adjusted life years (DALYs) at the global, regional, and national levels. At the global level, we examined trends in the incidence and prevalence of HL among populations aged 60 years and older, by sex and age. To control for age structure, we calculated age-standardized rates. At the regional level, we analyzed differences in the burden of HL among people aged 60 years or older in several different geographic regions. We explored the relationship between these differences and SDI. At the national level, we compared the burden of HL among people aged 60 years or older across 204 countries and regions, focusing on those in the high, medium-high, medium, medium-low, and low SDI quintiles, and assessed the burden of HL in these countries and regions.

### 2.3 Overall trend analysis

We assessed global trends in the dynamics of HL deaths, disability-adjusted life years (DALYs), and annual deaths over age 60 between 1990 and 2021. We explored trends in the burden of HL disease from the overall level to different regional levels. We used the Estimated Annual Percentage Change (EAPC) to illustrate global or regional trends in burden over a given period of time. EAPC’s assessment of ASR trends is a more reliable indicator for monitoring changes in the burden of disease because it is calculated using a linear regression model (20). The linear regression model is expressed as *y* = *α* + *βx*, where *y* = ln(ASR) and *x* = calendar year. The EAPC is calculated as (exp(β) − 1)×100%, and its 95% confidence interval (CI) is also derived from the model (21). The natural logarithm of the regression model was fitted through the time variable, and the natural logarithm of each observation was fitted to a straight line. The slope of the straight line is used to estimate the annual rate of change for a given period. Specifically, positive and negative values of EAPC, along with their 95% confidence intervals, indicate an upward or downward trend in disease burden.

### 2.4 J-J regression analysis

Joinpoint regression software version 4.9.1, developed by the National Cancer Institute, was used to analyze local trends in the burden of HL disease in people aged 60 years and older. Detailed methods for Joinpoint regression can be found on the software’s website^2^ and in the Li et al. (22). We used the Joinpoint regression model to divide the temporal trends in the burden of disease from 1990 to 2021 into consecutive, meaningful time periods. The analysis included annual percentage change (APC) and average annual percentage change (AAPC), along with corresponding 95% confidence intervals (CIs) to represent year-to-year (APC) and average (AAPC) trends in disease burden over a given time period (23). Specifically, positive or negative APC and AAPC values (*p* < 0.05) and their corresponding 95% confidence intervals indicate an increasing or decreasing trend in the burden of disease over a given period of time, respectively, whereas values close to zero indicate stabilization.

### 2.5 Age-period-cohort analysis

The contribution of age, period, and cohort effects to outcomes is of great significance to epidemiologic conclusions, but traditional methods have failed to overcome several shortcomings, including the inability to eliminate covariance between factors. Therefore, eigenestimation (IE) was then used to assess the effects of age, period, and cohort separately. The age-period-cohort model is a linear statistical method that can be used to demonstrate and analyze disease burden information, bypassing the previous method, which involved a multiclass model. Specifically, it can be illustrated with the following sentence: In (Refg) = *α* + Ae + Pf + Cg, where “Refg” denotes the incidence or mortality of PF in g birth cohorts, “e” refers to the age group, “f” stands for the period, and “Ae,” “Pf,” and “Cg” stand for the effects of age, period, and cohort, respectively.

Age, period, and cohort refer to the outcomes of population aging, objective and temporal variations in disease prevalence, and variations in outcomes among participants in the same birth cohort, respectively. Age, period, and cohort relative risks (RR) represent the relative risk of each age/period/cohort relative to the reference age/period/cohort, controlling for the other two dimensions. The RR is statistically significant when compared with 1. The RR is not statistically significant when compared with 1. For precise estimation, the data were recoded into consecutive 5-year age cohorts spanning the 5-year periods from 1990 through 2021, along with the corresponding 5-year birth cohorts. To determine the relative risk (RR) of a particular age, period, or birth cohort relative to the average portfolio level, we calculated these coefficients and aggregated them. This approach allows us to visualize the impact of different time dimensions on disease risk and provides a scientific basis for targeted prevention strategies. The systematic and comprehensive analysis provided by the age-period-cohort model provides stronger theoretical support for epidemiological studies, enabling us to accurately identify and address public health challenges.

### 2.6 Decomposition analysis

We used the decomposition analysis proposed by Das Gupta et al. (23), combined with the improved scheme developed by Cheng et al. (24), to examine the contributions to HL disability-adjusted life-years (DALYs) among individuals aged 60 years and older over the past three decades, considering population growth, population aging, and epidemiological changes. The contribution of changes in DALYs, py, ey = (ai,y * py * ei,y) for each region was calculated using the formula DALYs, py, ey = (ai,y * py * ei,y), following the method proposed by Xie et al. (25). The relative contribution of each variable to changes in DALYs in MAS populations was quantified by isolating the standardized effects of each multiplying factor. In addition, we decomposed DALY changes by gender and SDI groups to identify specific drivers of DALY changes in HL among people aged 60 years and older from 1990 to 2021.

### 2.7 Analysis of cross-country inequality

We used the Slope of Inequality Index (SII) and the Concentration Index (CI), which are able to show cross-national inequalities in HL burdens among people aged 60 years or older and are standardized metrics to quantify inequalities in HL burdens in many regions, allowing for a better narrowing of differences in the distribution of health and for further improvements in related policies, programs, and practices, as well as a comprehensive assessment of health inequalities. In this study, we compared data from 204 countries and territories over the period 1990 to 2021.

SII is a measure that uses regression modeling to represent the absolute difference in burden projections between those with the highest and lowest levels of SDI. Differences in regression-based estimates of health outcomes allow for the overall distribution of socio-economic factors to be taken into account. Positive/negative values of SII indicate the burden concentration in countries with higher/lower SDI levels. Absolute inequalities in health indicators can be quantified; the higher the absolute value, the greater the inequality.

The CI is a relative measure of inequality that quantifies the extent to which disadvantaged groups with low SDI burdens or advantaged groups with high SDI burdens are affected, and it concentrates the SDI to show a gradient in health across multiple subgroups in a natural ordering. It is derived by numerical integration under the Lorenz concentration curve, which is fitted to the cumulative percentage of DALYs relative to the population’s cumulative distribution by SDI (26). The health burden in low SDI countries is concentrated on the equality line under the Lorenz curve with a positive CI (27).

### 2.8 Predictive analysis

In this study, a Bayesian age-period-cohort (BAPC) modeling approach was used to further predict the burden of HL in terms of ASR, prevalence, and YLDs for people aged 60 years or older in the next 20 years, aiming to provide a scientific and reliable basis for decision-making by health organizations and researchers in response to future public health challenges. By accurately calculating marginal posterior distributions using the INLA method, the approach avoids the mixing and convergence issues associated with Markov chain Monte Carlo (MCMC) sampling, thereby improving the stability and efficiency of predictions (28). The theoretical basis and specific applications of various statistical analysis methods are detailed in Supplementary Table 1.

## 3 Results

### 3.1 Descriptive analysis of the disease burden of older adults HL at the global, regional, and national levels

Globally, between 1990 and 2021, the absolute number and age-standardized rates (ASRs) of disability among individuals aged 60 and above due to hearing loss (HL) have both significantly increased, with the estimated annual percentage change (EAPC) for disabilities caused by hearing loss slightly surpassing the prevalence (Tables 1, 2).

**Table 1:** Global and regional prevalence of older adults HL in 1990 and 2021, and EAPC of ASR from 1990 to 2021.

**Location**	**1990**		**2021**		**EAPC, 1990-2021**
	**Number**	**ASR**	**Number**	**ASR**	
Global	307785132.72 (277696062.60–340096582.10)	64494.30 (58179.35–71238.16)	728082818.34 (659506964.80–797637205.17)	67076.23 (60761.64–73484.87)	0.11 (0.1 to 0.12)
High SDI	83186191.79 (75222254.86–92035838.20)	57517.76 (52005.07–63616.20)	162628490.25 (146953737.99–179831527.95)	58181.26 (52581.34–64343.96)	0.02 (0 to 0.03)
High-middle SDI	79760528.89 (71888176.93–88249291.72)	64589.21 (58204.65–71424.44)	179043585.87 (161981761.49–194917992.35)	69812.13 (63172.10–76025.79)	0.26 (0.24 to 0.28)
Middle SDI	84653342.21 (75972083.54–93642623.12)	71953.21 (64590.73–79457.30)	240752368.87 (217079750.33–262405302.08)	73251.02 (66079.12–79803.81)	0.05 (0.03 to 0.06)
Low-middle SDI	44338563.79 (40247935.91–48970904.30)	65739.63 (59635.81–72665.72)	110218887.83 (100020779.26–122035697.20)	65666.83 (59564.70–72717.67)	–0.05 (–0.07 to –0.04)
Low SDI	15503950.04 (13997598.63–17231038.98)	62782.83 (56720.82–69680.29)	34827353.84 (31501980.25–38613275.50)	63204.37 (57209.56–69991.82)	0 (–0.03 to 0.04)
Andean Latin America	1280057.35 (1159071.10–1429154.63)	54737.80 (49510.32–61196.05)	3910856.85 (3536827.98–4371467.41)	54560.82 (49321.39–61016.92)	0.01 (0 to 0.02)
Australasia	1929639.44 (1814296.92–2045579.05)	62429.64 (58659.54–66198.63)	4608891.01 (4117999.78–5097520.55)	64310.78 (57419.59–71222.73)	0.14 (0.08 to 0.2)
Caribbean	1928587.54 (1727550.33–2173058.57)	60609.95 (54269.88–68292.95)	4063968.00 (3646465.64–4574950.10)	60406.56 (54203.65–68002.27)	0 (–0.01 to 0)
Central Asia	3403652.45 (3062018.69–3807328.07)	61694.41 (55459.02–69123.26)	5848297.22 (5269270.60–6510095.03)	61906.15 (55701.24–69092.09)	0.01 (0.01 to 0.01)
Central Europe	11901145.53 (10712273.74–13326990.36)	62104.75 (55886.85–69580.41)	18821656.07 (16932162.75–21058665.27)	62212.82 (55977.36–69562.79)	0.01 (0 to 0.01)
Central Latin America	5750052.29 (5163424.42–6468376.91)	61056.27 (54782.23–68749.99)	18636043.95 (16709296.20–20941736.66)	60859.81 (54540.83–68442.34)	0 (0 to 0)
Central Sub-Saharan Africa	1301451.86 (1182115.74–1428459.45)	54873.65 (49932.40–60292.89)	3023110.49 (2749495.16–3323419.74)	54380.02 (49538.82–59786.64)	–0.02 (–0.04 to –0.01)
East Asia	80019094.01 (70918847.30–89314118.32)	78398.93 (69559.03–87141.20)	229663559.36 (204845859.09–249156202.34)	82407.73 (73568.67–89429.59)	0.17 (0.14 to 0.19)
Eastern Europe	22358156.43 (20139223.84–24994531.18)	62324.67 (56098.45–69722.60)	29946987.08 (27019878.68–33446845.41)	62564.71 (56436.30–69923.98)	0.01 (0.01 to 0.01)
Eastern Sub-Saharan Africa	5759132.61 (5021499.77–6601121.57)	70754.42 (61824.87–80672.13)	12976385.21 (11336010.70–14742295.49)	72344.97 (63333.16–81827.21)	0.07 (–0.05 to 0.19)
High-income Asia Pacific	14354689.36 (13025869.85–15960842.31)	57450.90 (52084.21–63903.43)	35588225.36 (32113617.59–39704293.96)	57584.65 (52095.88–64073.98)	0.02 (0.01 to 0.02)
High-income North America	29993870.09 (26543936.14–33573685.68)	63882.12 (56542.33–71534.97)	54643654.51 (48596691.54–61256314.53)	61402.40 (54607.52–68827.59)	–0.12 (–0.15 to –0.09)
North Africa and Middle East	10748766.46 (9750434.01–11885760.74)	58717.76 (53222.85–64974.57)	29244228.15 (26494095.66–32304046.50)	58551.59 (53014.35–64719.38)	–0.01 (–0.01 to –0.01)
Oceania	228995.56 (202300.74–258280.67)	73110.45 (64613.88–82296.58)	575905.42 (509293.68–650895.00)	74070.15 (65526.98–83596.91)	–0.05 (–0.07 to –0.02)
South Asia	41437072.65 (37732188.33–45687374.96)	66527.92 (60501.73–73510.39)	116280783.25 (105944389.67–128441449.76)	66557.69 (60583.86–73583.86)	–0.07 (–0.09 to –0.05)
Southeast Asia	21439258.31 (19225796.16–23904070.24)	75212.45 (67418.37–83780.30)	59380982.26 (53031335.27–66377022.52)	75975.06 (67838.92–84813.85)	–0.04 (–0.07 to –0.02)
Southern Latin America	3258456.07 (2917634.53–3642306.30)	56073.42 (50162.56–62722.63)	6345612.54 (5692042.33–7106770.99)	55996.80 (50242.05–62686.43)	0 (–0.01 to 0)
Southern Sub-Saharan Africa	1586457.48 (1459836.54–1725478.41)	51020.63 (46938.47–55536.46)	3433944.43 (3170105.16–3719403.83)	51295.36 (47339.49–55606.30)	0.02 (0 to 0.03)
Tropical Latin America	7219418.39 (6429304.25–8135156.17)	68694.40 (61151.76–77324.97)	21893341.53 (19505824.35–24677572.06)	68402.82 (60935.47–77075.39)	–0.01 (–0.03 to 0)
Western Europe	36870888.08 (33611462.50–40498858.74)	47954.79 (43729.65–52633.85)	59137981.47 (53832290.29–65026599.73)	47842.98 (43600.14–52514.20)	–0.05 (–0.07 to –0.04)
Western Sub-Saharan Africa	5016290.76 (4569817.54–5491741.85)	50665.27 (46257.99–55377.93)	10058404.17 (9177385.78–10987651.94)	48135.94 (44042.39–52477.23)	–0.13 (–0.21 to –0.05)

**Table 2:** Global and regional YLDs of older adults HL in 1990 and 2021, and EAPC of ASR from 1990 to 2021.

**Location**	**1990**		**2021**		**EAPC, 1990-2021**
	**Number**	**ASR**	**Number**	**ASR**	
Global	10915444.45 (7162134.01–15938048.85)	2383.17 (1577.44–3457.39)	26590896.41 (17574938.84–38477291.58)	2483.93 (1647.18–3586.65)	0.17 (0.15 to 0.19)
High SDI	2916903.13 (1917594.45–4253004.05)	2017.90 (1326.35–2942.32)	5833912.12 (3846849.74–8486436.27)	2019.24 (1322.41–2951.38)	0.07 (0.05 to 0.09)
High-middle SDI	2772893.04 (1809154.11–4056571.92)	2357.95 (1553.67–3424.39)	6484974.04 (4257609.23–9375829.53)	2556.41 (1682.42–3690.99)	0.31 (0.28 to 0.35)
Middle SDI	2909063.35 (1894712.01–4264845.03)	2653.12 (1755.72–3845.06)	8713576.98 (5727354.37–12586508.19)	2741.86 (1816.47–3941.32)	0.14 (0.11 to 0.16)
Low-middle SDI	1683176.82 (1108852.17–2458108.93)	2658.85 (1778.45–3840.45)	4151994.87 (2745173.85–6044455.49)	2587.11 (1728.99–3738.00)	–0.11 (–0.12 to –0.09)
Low SDI	621248.76 (407056.98–907429.87)	2711.78 (1809.69–3909.21)	1384113.02 (910532.96–2012033.44)	2667.84 (1780.00–3841.91)	–0.05 (–0.09 to –0.01)
Andean Latin America	44020.60 (28519.15–65150.45)	1944.92 (1266.53–2866.99)	136311.77 (88450.27–200997.44)	1924.46 (1251.06–2833.56)	0.05 (0.01 to 0.09)
Australasia	64957.22 (43129.96–93850.57)	2130.18 (1417.73–3073.24)	157281.89 (101873.24–231982.90)	2142.87 (1380.78–3172.46)	0.21 (0.11 to 0.31)
Caribbean	69071.44 (44872.30–101791.03)	2221.94 (1450.61–3262.90)	148317.56 (96710.03–217997.31)	2194.17 (1429.19–3226.74)	–0.01 (–0.02 to 0)
Central Asia	121071.82 (78347.81–178420.82)	2275.53 (1480.87–3339.92)	199568.41 (128965.56–295103.90)	2258.52 (1479.35–3311.15)	0.01 (0 to 0.03)
Central Europe	423823.85 (276176.16–625570.87)	2297.19 (1508.23–3372.67)	695578.58 (455405.04–1021500.16)	2276.37 (1488.31–3346.20)	0.03 (0 to 0.05)
Central Latin America	200923.06 (130530.82–296612.90)	2223.67 (1456.29–3262.60)	660786.85 (430776.02–974302.04)	2200.57 (1440.08–3235.34)	0 (–0.01 to 0.02)
Central Sub-Saharan Africa	52982.08 (34125.35–78631.98)	2477.46 (1636.36–3603.16)	123245.21 (80377.06–181170.52)	2426.03 (1611.15–3510.38)	–0.05 (–0.07 to –0.03)
East Asia	2565487.44 (1648743.22–3786921.89)	2737.57 (1790.04–3986.80)	8128700.53 (5319147.66–11734554.02)	2993.51 (1969.53–4304.75)	0.37 (0.31 to 0.42)
Eastern Europe	823919.11 (541623.28–1206020.74)	2387.01 (1579.08–3476.38)	1116071.38 (733676.15–1626809.91)	2362.59 (1557.42–3439.11)	0 (–0.02 to 0.01)
Eastern Sub-Saharan Africa	239544.77 (153490.65–352741.89)	3180.22 (2078.67–4614.05)	540217.49 (347176.66–795032.05)	3215.53 (2099.10–4674.57)	0.04 (–0.09 to 0.16)
High-income Asia Pacific	454548.73 (294967.27–676204.29)	1874.71 (1223.47–2775.38)	1241117.24 (818855.51–1816520.62)	1849.60 (1201.76–2740.45)	0.06 (0.02 to 0.1)
High-income North America	1146700.91 (752164.70–1670576.23)	2425.45 (1587.65–3539.30)	2070072.03 (1363965.63–3017819.20)	2310.08 (1520.38–3371.07)	–0.11 (–0.16 to –0.06)
North Africa and Middle East	445545.78 (296020.45–646194.78)	2567.33 (1730.00–3688.54)	1174212.83 (776042.80–1716493.43)	2451.44 (1638.52–3556.08)	–0.12 (-0.13 to -0.11)
Oceania	6987.74 (4408.89–10495.60)	2518.88 (1635.15–3708.92)	17784.52 (11296.54–26602.86)	2523.50 (1638.39–3719.27)	-0.03 (–0.05 to –0.02)
South Asia	1582126.07 (1043564.82–2304810.57)	2724.31 (1825.91–3919.06)	4408016.69 (2927911.99–6388187.16)	2639.74 (1771.30–3798.08)	–0.13 (–0.15 to –0.12)
Southeast Asia	756442.42 (492752.18–1113749.96)	2819.57 (1864.63–4110.67)	2064137.34 (1345414.89–3046923.35)	2792.83 (1846.57–4085.99)	–0.08 (–0.1 to –0.06)
Southern Latin America	114690.65 (74516.83–168071.15)	2035.43 (1330.73–2969.10)	229115.78 (149446.64–339150.14)	2002.04 (1303.33–2967.46)	–0.02 (–0.03 to 0)
Southern Sub-Saharan Africa	57152.25 (37293.54–84060.33)	1935.31 (1275.77–2823.39)	120298.01 (78445.98–175660.19)	1920.33 (1267.66–2777.98)	0 (–0.03 to 0.02)
Tropical Latin America	253771.29 (165196.42–374638.41)	2539.64 (1673.77–3714.97)	790064.42 (516411.51–1155934.43)	2508.11 (1645.62–3659.57)	–0.04 (–0.08 to 0)
Western Europe	1276275.83 (835676.42–1876375.88)	1642.01 (1073.31–2415.57)	2145111.62 (1410240.02–3120727.24)	1624.82 (1056.06–2383.32)	0.05 (0.02 to 0.09)
Western Sub-Saharan Africa	215401.40 (141379.00–312470.80)	2289.37 (1523.37–3287.61)	424886.25 (278016.21–615891.26)	2147.77 (1424.83–3080.40)	–0.13 (–0.21 to –0.06)

**Figure 1:**
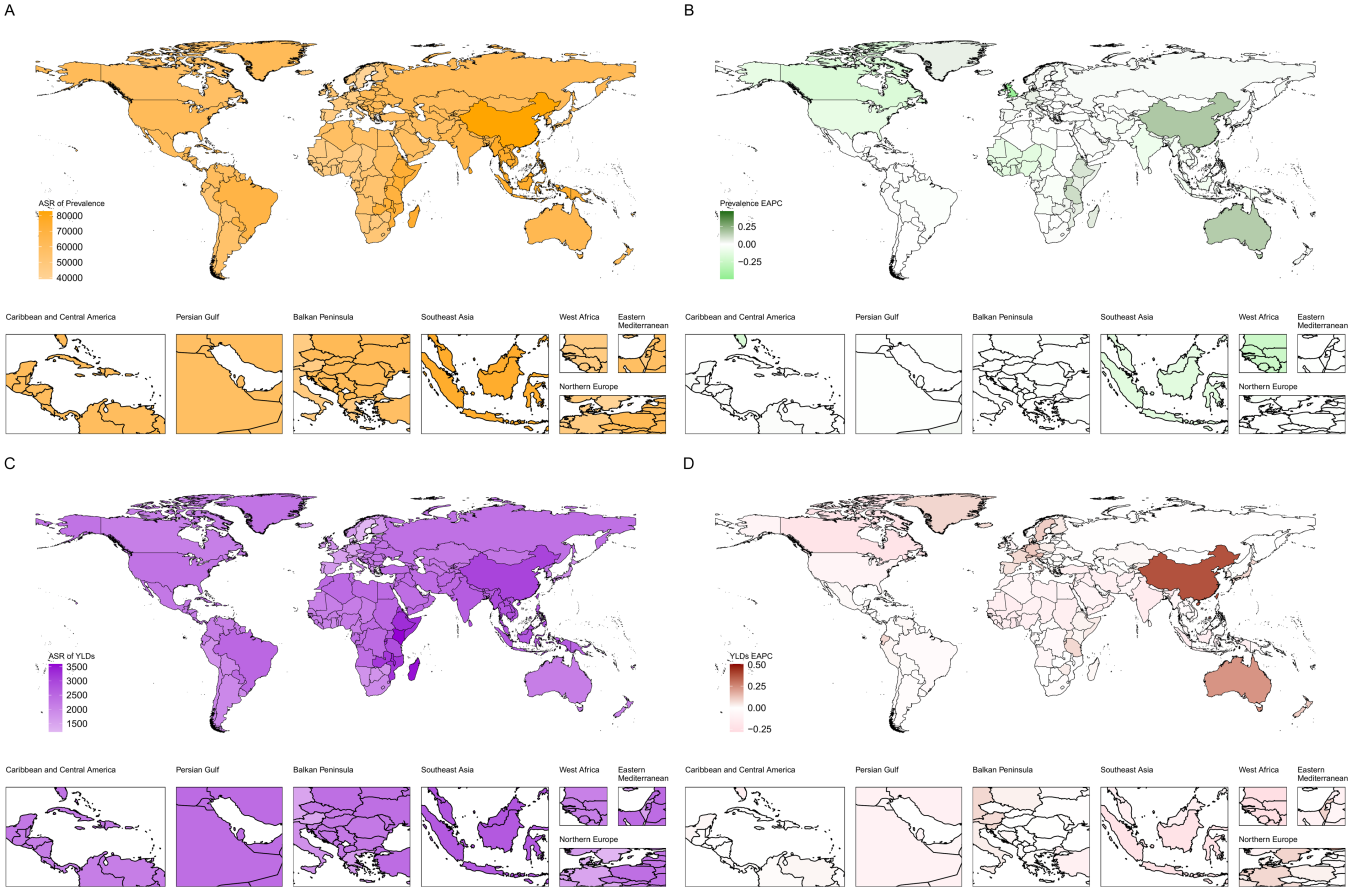
Descriptive analysis of the disease burden of older adults HL at the global, regional, and national levels **(A)**: Global and regional age-standardized rate (ASR) of hearing loss prevalence among adults aged ≥60 years, 1990-2021; **(B)**: Estimated annual percentage change (EAPC) of hearing loss prevalence among adults aged ≥60 years by region, 1990-2021; **(C)**: Global and regional age-standardized rate (ASR) of hearing loss-related YLDs among adults aged ≥60 years, 1990-2021; **(D)**: Estimated annual percentage change (EAPC) of hearing loss-related YLDs among adults aged ≥60 years by region, 1990-2021).

From a regional perspective, we observed significant differences in the burden indicators across social demographic index (SDI) quintiles and geographical areas in 2021 (Figure 1). In terms of the absolute number of cases, the burden is highest in the median SDI quintile and lowest in the low SDI quintile. In contrast, when age-standardized rates are considered, the prevalence in high SDI quintiles shows the highest age-standardized rate (ASR), while the prevalence in low SDI quintiles is the lowest. The incidence rate among the top fifth of the population with high SDI has shown the largest increase over time (measured by EAPC), while the incidence rate among the bottom fifth of the population with low SDI has increased the least.

Geographically, East Asia has consistently had the highest absolute numbers of cases and YLDs. In contrast, Western Europe has the lowest age-standardized incidence rate (ASR), while Eastern sub-Saharan Africa has the highest YLD ASR. South Asia (incidence) and East Asia (YLDs) show the most significant upward trends in ASRs, while Western Europe and southern Latin America have shown stable or slightly declining trends during the study period.

Nationally, the patterns reflected these regional disparities. China recorded the highest absolute number of prevalent cases and YLDs, while Kenya had the highest ASR for YLDs. At the other end of the spectrum, countries such as Sweden and the United Kingdom were among the lowest ASR countries. A comprehensive listing of national-level data for all 204 countries and territories is provided in Supplementary Tables S2, S3.

### 3.2 Current overall trend analysis of the disease burden of older adults HL

**Figure 2:**
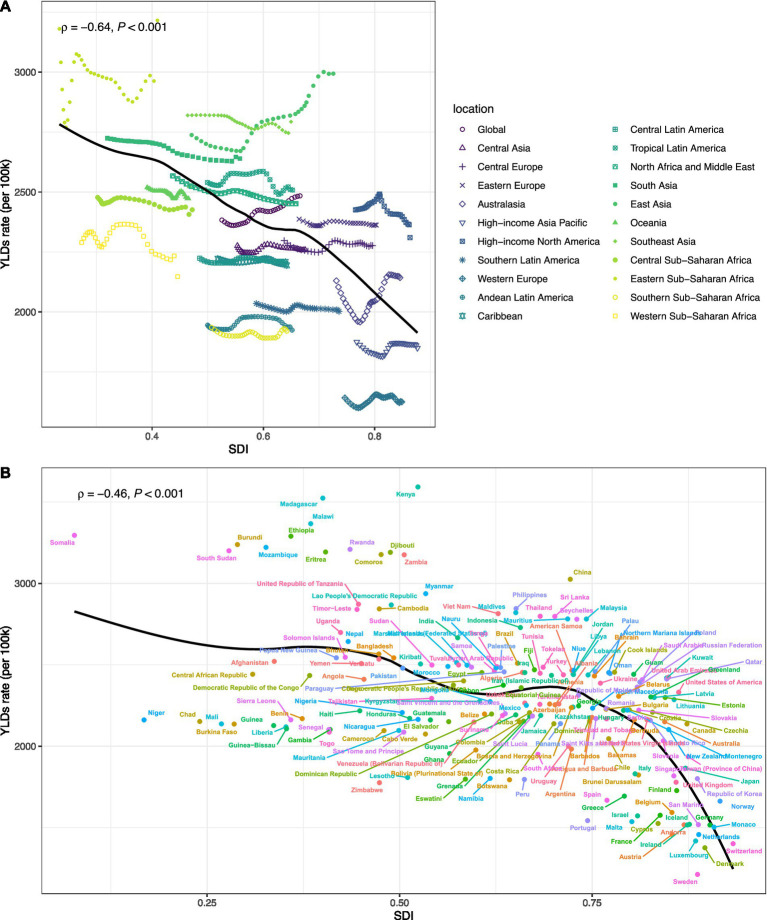
Current overall trend analysis of the disease burden of older adults HL **(A)**: Correlation between Socio-Demographic Index (SDI) and age-standardized YLDs rate of hearing loss among adults aged ≥60 years at the regional level, 1990-2021; **(B)**: Correlation between Socio-Demographic Index (SDI) and crude YLDs rate of hearing loss among adults aged ≥60 years at the national level, 1990-2021).

The results in Figure 2 show that the overall trend from 1990 to 2021 indicates a continuous increase in the burden of older adults HL across all indicators, with distinct demographic characteristics. The absolute number of cases has grown most significantly, primarily influenced by global population growth and aging. The relationship between national SDI and HL burden presents a complex non-linear pattern. Overall, as SDI increases, the ASR of YLD declines. However, many regions significantly deviate from this general pattern. For example, East Asia, South Asia, and East Sub-Saharan Africa lie above the smoothed curve, indicating that their burden is higher than what is expected based solely on their SDI levels. In contrast, Western Europe is well below the expected value, indicating that its burden is lower than anticipated. This underscores that, in addition to overall socio-economic development, specific factors such as environmental exposures or healthcare policies also play a crucial role in determining HL burden.

In addition, it is worth mentioning that gender differences are evident across all indicators. Throughout the study period, the crude and age-standardized incidence rates (ASRs) of men consistently exceeded those of females in terms of prevalence and disease burden (YLDs). However, due to the generally longer lifespan of females, the absolute number of cases is highest among the women aged 60 and above group, followed by males, and then the combined group of both sexes (‘bBoth’) (Supplementary Figure 2).

### 3.3 J-J regression analysis was used to analyze the local trend of the disease burden of older adults HL

**Figure 3:**
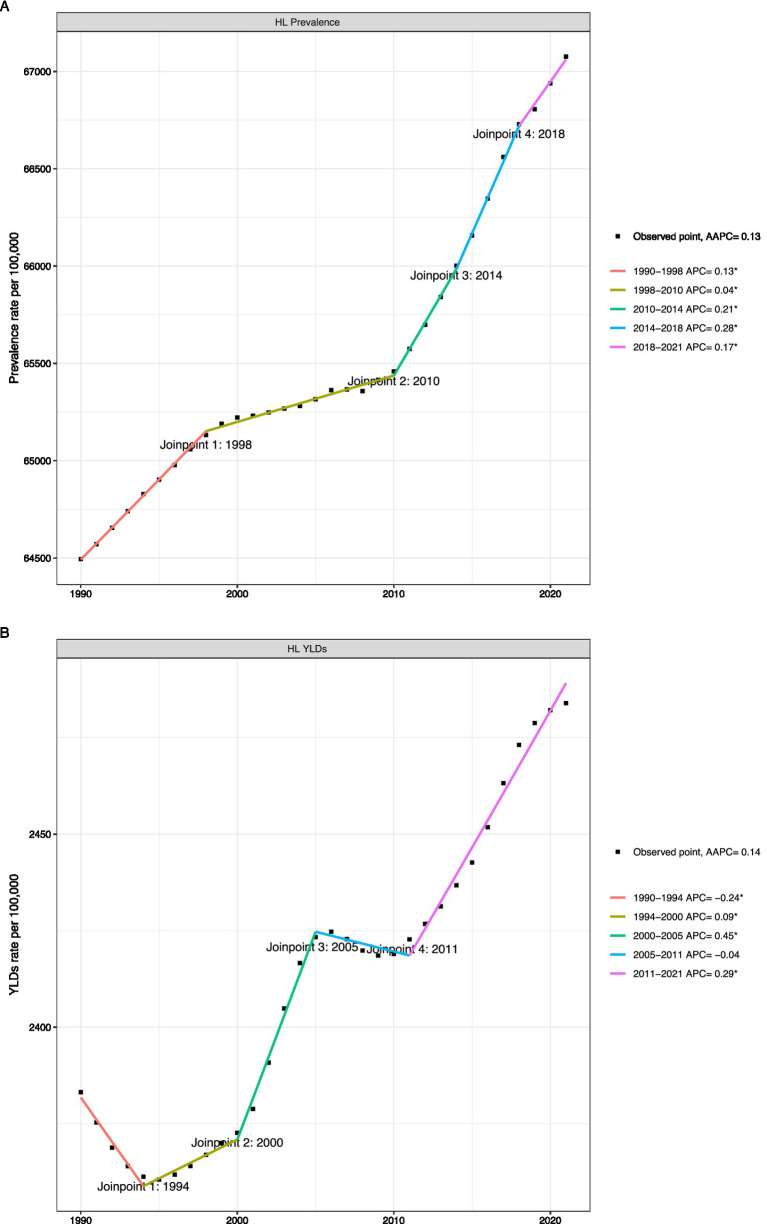
Local trend of disease burden of older adults HL **(A)**: Joinpoint regression analysis of age-standardized prevalence rate of hearing loss among adults aged ≥60 years, 1990-2021; **(B)**: Joinpoint regression analysis of age-standardized YLDs rate of hearing loss among adults aged ≥60 years, 1990-2021).

The Joinpoint regression analysis revealed specific temporal inflection points in the trend of HL burden, which are not evident in the overall EAPC (Figure 3A). In terms of the age-standardized prevalence rate (ASR), the entire period shows a steady increase (AAPC = 0.13). However, this trend is not consistent: there was a particularly rapid increase from 2014 to 2018 (APC = 0.28).

The trend of YLDs’ ASR is more dynamic, resembling a ‘W’-shaped curve. Significant declines occurred from 1990 to 1994 (APC = −0.24) and from 2005 to 2011 (APC = −0.04), while the fastest growth occurred from 2000 to 2005 (APC = 0.45). This non-linear trajectory indicates that changes in YLDs are influenced by the interaction of various complex factors, including changes over time in disability weights, diagnostic practices, and treatment availability (Figure 3B).

### 3.4 Age-period-cohort analysis on older adults HL prevalence

**Figure 4:**
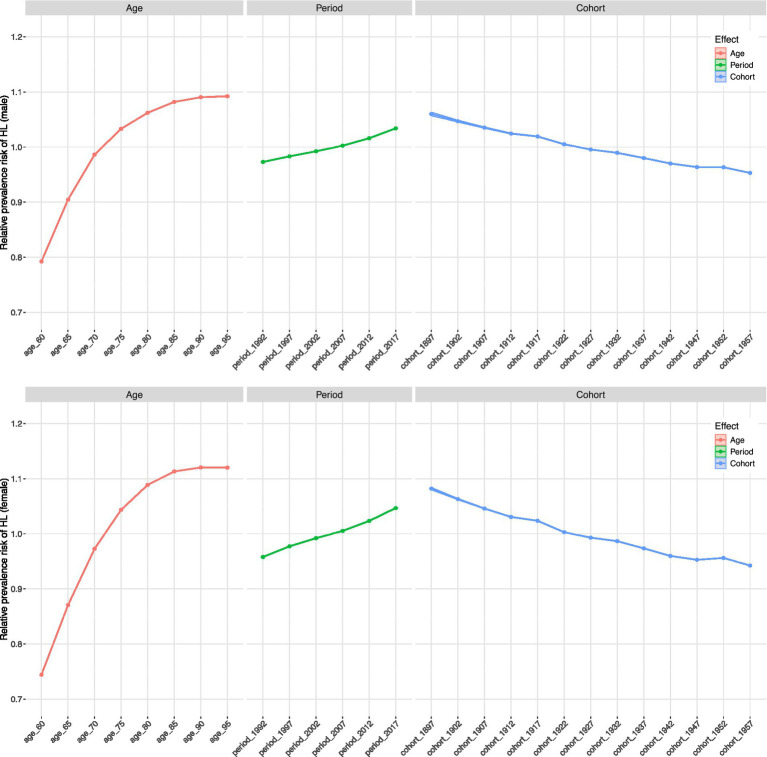
Age-period-cohort analysis on older adults HL prevalence.

Age-period-cohort (APC) analysis shows that age, calendar period, and birth cohort have independent effects on the risk of hearing loss (HL) (Figure 4 and Table 3). Unsurprisingly, the impact of age is greatest, with relative risks (RRs) increasing monotonically with age. The rate of increase slows after age 85. The period effect shows that from 1990 to 2021, RRs have gradually but steadily increased, indicating that the influencing factors affect all age groups simultaneously, such as improvements in detection and diagnosis or changes in environmental risk factors. In contrast, a significant birth-cohort effect was observed; after controlling for age and period, the risk of HL decreased across successive birth cohorts born after the early 20th century. This suggests that improvements in childcare and nutrition, or a reduction in early life exposure to ototoxic factors, may have a protective effect on later-born populations. The age-stratified prevalence RRs for hearing loss by gender are shown in Supplementary Table 4. For details of the age-period-cohort analysis model, see Supplementary Figure 3.

**Table 3:** RRs of older adults HL prevalence for sexes due to age, period and birth cohort effects.

**Factor**	**Prevalence**	
	**RR (95% CI)**	***P***
**Age (years)**		
60–64	0.771(0.771–0.772)	<0.001
65–69	0.890(0.889–0.890)	<0.001
70–74	0.980(0.980–0.981)	<0.001
75–79	1.038(1.038–1.038)	<0.001
80–84	1.075(1.075–1.075)	<0.001
85–89	1.096(1.096–1.096)	<0.001
90–94	1.102(1.102–1.103)	<0.001
95–99	1.103(1.102–1.103)	<0.001
**Period**		
1992	0.965(0.965–0.965)	<0.001
1997	0.980(0.980–0.980)	<0.001
2002	0.992(0.992–0.992)	<0.001
2007	1.004(1.004–1.004)	<0.001
2012	1.020(1.020–1.020)	<0.001
2017	1.041(1.041–1.041)	<0.001
**Birth cohort**		
1897	1.069(1.068–1.071)	<0.001
1902	1.054(1.053–1.055)	<0.001
1907	1.040(1.039–1.040)	<0.001
1912	1.027(1.026–1.027)	<0.001
1917	1.021(1.021–1.022)	<0.001
1922	1.004(1.003–1.004)	<0.001
1927	0.995(0.995–0.995)	<0.001
1932	0.989(0.989–0.990)	<0.001
1937	0.978(0.978–0.978)	<0.001
1942	0.966(0.966–0.966)	<0.001
1947	0.959(0.959–0.959)	<0.001
1952	0.960(0.960–0.960)	<0.001
1957	0.947(0.947–0.947)	<0.001

### 3.5 Decomposition analysis of older adults HL

**Figure 5:**
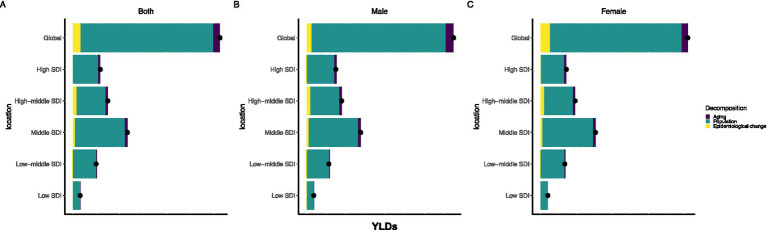
Decomposition analysis about older adults HL **(A)**: Decomposition of changes in hearing loss-related YLDs among adults aged ≥60 years by demographic and epidemiological factors (Both genders), 1990-2021; **(B)**: Decomposition of changes in hearing loss-related YLDs among adults aged ≥60 years by demographic and epidemiological factors (Male), 1990-2021; **(C)**: Decomposition of changes in hearing loss-related YLDs among adults aged ≥60 years by demographic and epidemiological factors (Female), 1990-2021).

Globally, over a 30-year research period, the total increase in YLDs caused by hearing loss was primarily driven by population growth (90.25%) and aging of the population (4.64%), while changes in epidemiological incidence accounted for only a small proportion (5.12%) (Figure 5 and Supplementary Table 5). However, this pattern varies significantly by SDI. In high- and upper-middle SDI regions, the epidemiological change component is negative, indicating that the underlying risk of HL has increased (decreased) over time, which is offset by demographic factors. In low- and lower-middle SDI regions, the impact of population growth is greatest. Notably, in upper-middle SDI quintile regions, the impact of epidemiological changes (despite being negative) is proportionally greater than in other quintile regions, suggesting that these areas are in a transitional phase in which public health interventions may already be positively affecting HL risk factors. The breakdown analysis by sex reflects these overall patterns. The changes in years lived with disability due to hearing loss among older adults are detailed in Supplementary Table 5.

### 3.6 Correlation between SDI and disease and cross-country inequality analysis of older adults HL

**Figure 6:**
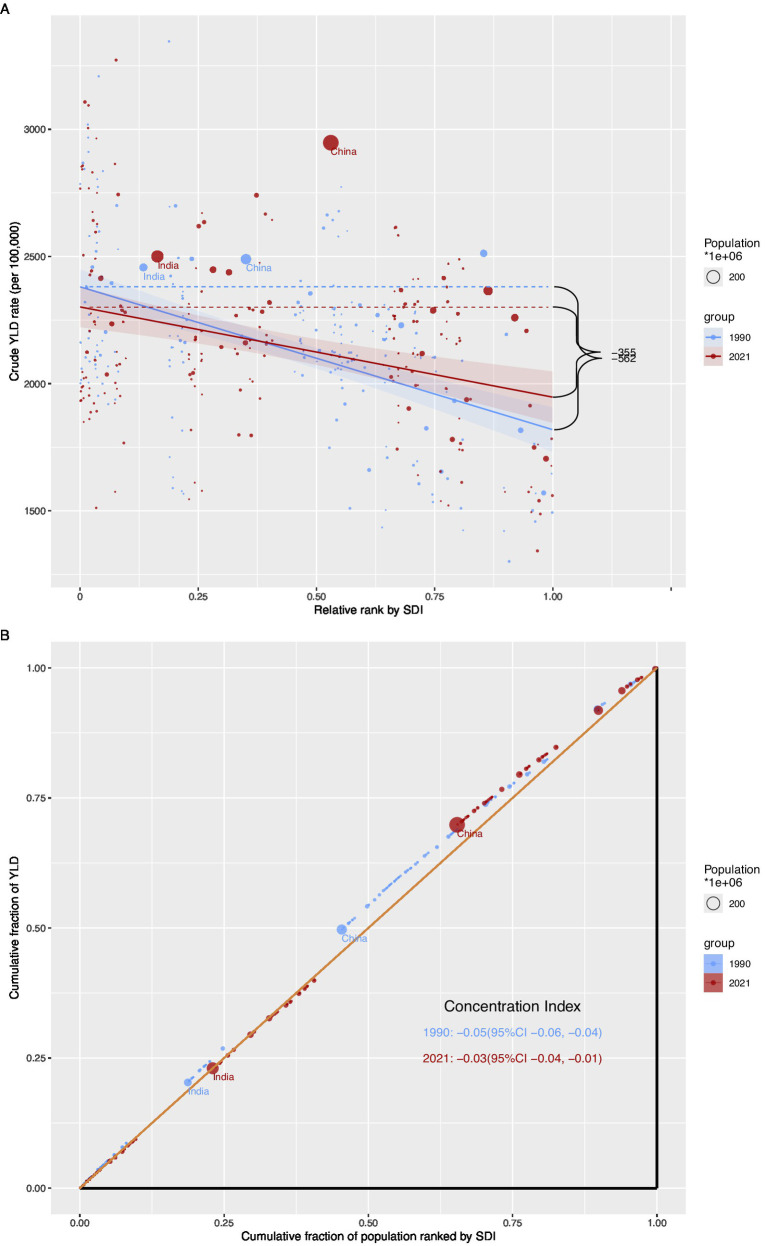
Correlation between SDI and disease and cross-country inequality analysis of older adults HL **(A)**: Slope Index of Inequality (SII) for crude YLDs rate of hearing loss among adults aged ≥60 years across countries, 1990 and 2021; **(B)**: Concentration Index (CI) for crude YLDs rate of hearing loss among adults aged ≥60 years across countries, 1990 and 2021).

Inequality analysis confirms that a significant transnational socioeconomic gradient in HL burden persisted throughout the study period, although there was a trend of narrowing (Figure 6). Between 1990 and 2021, the inequality slope index (SII) and concentration index (CI) of crude YLD rates and prevalence were both negative. This indicates that the burden of HL has been concentrated in countries with lower SDI. However, the degree of inequality has decreased over time. For instance, the SII of the crude YLD rate dropped from −562.06 per 100,000 (95% UI: −706.34, −417.79) in 1990 to −354.79 (95% UI: −496.07, −213.51) in 2021. This convergence suggests that, while the burden in low-income countries remains disproportionate, the gap between high- and low-SDI countries has been narrowing, which may be attributed to economic development and improvements in healthcare services across many regions of the world.

### 3.7 Predictive analysis of older adults HL to 2040

**Figure 7:**
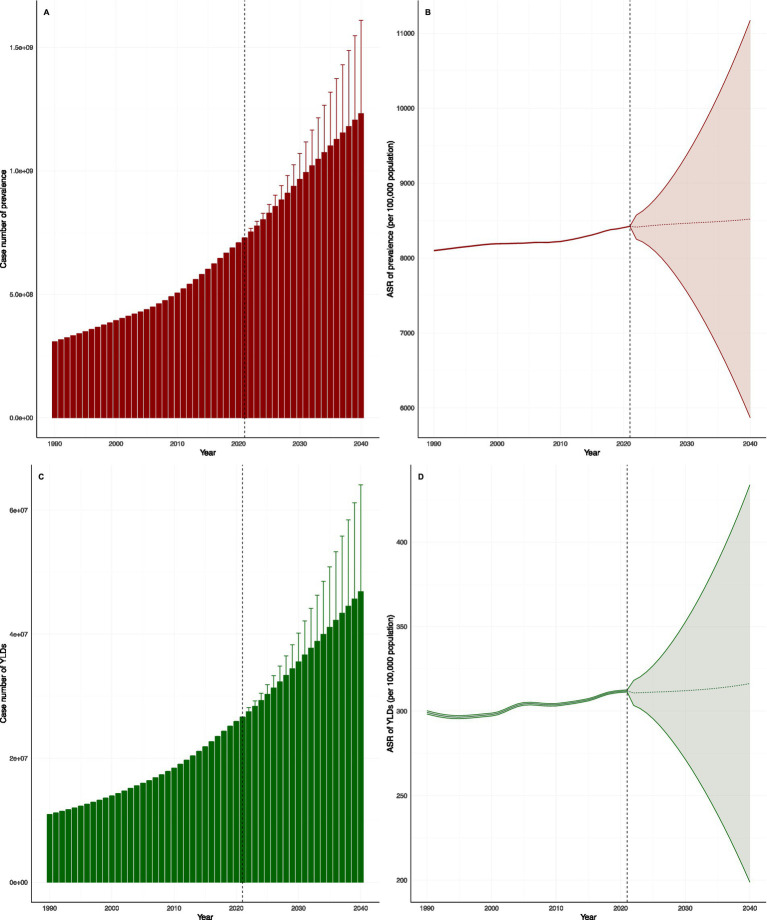
Predictive analysis of older adults HL to 2040 **(A)**: Predicted number of hearing loss prevalent cases among adults aged ≥60 years globally, 1990-2040; **(B)**: Predicted age-standardized rate (ASR) of hearing loss prevalence among adults aged ≥60 years globally, 1990-2040; **(C)**: Predicted number of hearing loss-related YLDs cases among adults aged ≥60 years globally, 1990-2040; **(D)**: Predicted age-standardized rate (ASR) of hearing loss-related YLDs among adults aged ≥60 years globally, 1990-2040).

According to the Bayesian age-period-cohort model, the burden of older adults HL is expected to continue to rise until 2040 (Figure 7). This is primarily due to the anticipated global population growth and aging, with the absolute number of prevalent cases and YLD expected to surge dramatically. For instance, the number of YLD is projected to increase from 27.5 million in 2022 to 46.9 million in 2040. In contrast, age-standardized rates (ASRs) are expected to remain relatively stable, with only a slight increase. This disparity highlights that future challenges in managing HL will be driven more by changes in population structure than by worsening age-specific risks. Supplementary Table 6 provides detailed predictions for the data above.

## 4 Discussion

Studying the global burden of hearing loss in older adults is crucial due to its prevalence and profound impact on health outcomes and quality of life. Hearing loss ranks as the third most common cause of years lived with disability (YLD) worldwide, particularly affecting individuals over the age of 70, where it becomes the leading cause of YLD in this demographic (18). The World Health Organization (WHO) emphasizes that hearing loss can lead to significant communication difficulties, which, in turn, affect healthcare utilization and overall satisfaction with health services. Furthermore, the economic burden of unaddressed hearing impairment is considerable, with estimated annual costs reaching up to $980 billion due to healthcare expenses, lost productivity, and educational support needs (29, 30). The effects of hearing loss extend beyond communication challenges; it is associated with a range of adverse health outcomes, including cognitive decline, depression, and an increased risk of dementia (31, 32). Therefore, global attention and research on hearing loss in older adults are essential not only to enhance individuals’ quality of life but also to reduce the socioeconomic burden and improve overall public health.

This study used GBD 2021 data to systematically analyze the global burden of hearing loss among individuals aged 60 and above from 1990 to 2021. The main findings include: (1) The prevalence and age-standardized rates (ASR) of hearing loss in older adults are showing a significant upward trend globally, with huge regional inequalities; the absolute burden is heaviest in East Asia, while Western Europe has the lowest age-standardized rates; (2) There is a complex non-linear relationship between disease burden and the Socio-Demographic Index (SDI); (3) There are significant gender differences in disease burden, with men experiencing a higher burden than women; (4) The increase in burden is mainly attributed to population growth and aging, rather than changes in epidemiological risks; (5) Although there is cross-national health inequality, it is narrowing; and (6) Predictions indicate that the disease burden will continue to increase significantly by 2040.

The burden of hearing loss varies considerably across different regions, closely correlating with socioeconomic factors (1, 33). Study results indicate that countries with a low socio-demographic index (SDI) experience a higher burden of hearing loss, consistent with previous research (34, 35). Socioeconomic factors are pivotal in influencing the prevalence of hearing loss; economic status not only affects individuals’ behavior in seeking medical assistance but also determines their access to essential hearing care services (36). Countries and regions with higher SDI possess distinct advantages in alleviating the burden of hearing loss, attributable to more comprehensive occupational noise control regulations, the widespread use of protective equipment, adequate resources for otolaryngologists, and sufficient financial support (37, 38). Furthermore, hearing aids are more readily available in these areas, contributing positively to the reduction of hearing loss burden among older adults (39). Some countries offer hearing aids to older adults through public programs, further mitigating the disease burden associated with age-related hearing loss (ARHL) (40). However, even in the UK, where the National Health Service provides bilateral hearing aids, uptake remains low (41), indicating that access and affordability are not the sole barriers hindering the utilization of hearing care services. Early detection of hearing loss and timely intervention are essential for mitigating its impact on quality of life (42). In high-income countries, such as those in North America and Europe, the burden of hearing loss has decreased due to heightened awareness, widespread screening, and the implementation of rehabilitation interventions (43). However, data regarding hearing loss and rehabilitation interventions in low- and middle-income countries remain scarce (1, 44). Noise exposure, particularly occupational noise exposure, is a significant risk factor for hearing loss (45). Consequently, individuals with lower levels of education and socioeconomic status are more likely to be employed in occupations with higher noise exposure, leading to a greater prevalence of hearing loss in these populations (33, 46). Patients in low- and middle-income countries in Latin America often encounter inadequate public health policies (46–49), and the use of hearing aids is generally low in these regions (50, 51). Therefore, this preliminary evidence underscores the necessity for hearing care campaigns in low- and middle-income countries to enhance diagnosis, improve access to treatment, and facilitate subsequent rehabilitation processes for these populations. This also indicates significant disparities in hearing aid utilization.

Through cross-country inequality analysis, we find that the disparity between low- and high-income groups has narrowed significantly, likely due to historical efforts to achieve this outcome. To mitigate the impact of occupational noise on hearing loss, various measures have been implemented, including the use of protective equipment, the formulation of regulations, and supervision (52). A clear downward trend in OSHA occupational noise exposure measurements was observed from 1979 to 2013 (53). In recent years, some scholars have begun advocating for hearing screening for older adults (54). In 2012, the US Preventive Services Task Force issued guidelines for hearing screening in individuals aged 50 and older, detailing risk assessment, screening tests, interventions, and the balance of benefits and harms (55). Advances in hearing screening have enabled the identification of many patients with mild age-related hearing loss (ARHL). Previous research has indicated that malnutrition is a potential risk factor for hearing loss (56). Economic growth in certain regions may improve residents’ diets, thereby reducing the incidence of hearing loss (57–59).

Furthermore, improvements in the global economy have increased individuals’ purchasing power (60), enabling more people to invest in medical services that were previously unaffordable, such as hearing aids and cochlear implants (40). The improvement in economic conditions allows individuals to seek professional help for hearing loss rather than suffering in silence, thereby increasing the likelihood of identifying mild hearing loss. Collectively, these measures and advancements have contributed to global mitigation of hearing loss and the reduction of health inequalities across income groups.

Crucially, our age-period-cohort analysis provides deeper temporal insights: across all birth cohorts and calendar periods studied, men exhibited higher, consistently stable relative risks (RRs). This indicates that men’s disadvantage in hearing health is a persistent, stable phenomenon that has not diminished across generations born throughout the 20th century.

This enduring gap likely stems from the combined effects of multiple factors that have remained unchanged over time. First, consistent with prior literature, age-related degeneration of the auditory system is more pronounced in males (61). Second, biological mechanisms, such as potential differences in mitochondrial DNA damage, may also contribute to this inherent inequality (62, 63). Furthermore, males have historically been exposed to greater occupational noise, a major risk factor for hearing loss (64). Men typically exhibit higher hearing thresholds in the high-frequency range, potentially contributing to their higher incidence of hearing loss compared to women (65). Furthermore, men are generally less likely to seek help for hearing issues proactively. Our APC model reveals the persistence of this gap, indicating that public health and occupational safety initiatives to date have been insufficient to narrow the gender disparity in hearing health. This highlights an area requiring targeted future interventions.

Our projections indicate that as the global population ages, both years of disability (YLD) and the prevalence of hearing loss, including age-standardized rates (ASR), will continue to rise. This trend is primarily attributed to significant changes in the world’s demographic structure: the number of individuals aged 60 and above is anticipated to increase from approximately 970 million in 2019 to approximately 2.1 billion by 2050 and further to 3.1 billion by 2100 (66). This phenomenon of population aging is particularly pronounced in developing countries, where it is expected that around 80% of the aging population will reside (67). As the older population expands, the economic and social costs associated with hearing loss will also rise markedly. These costs encompass healthcare expenditures, lost productivity, and social services (68). Consequently, the demand for hearing services is projected to increase significantly, potentially exerting considerable pressure on the existing healthcare system (69). To address these challenges, it is essential to prioritize hearing health and develop effective strategies to enhance access to hearing care for older adults. By implementing these measures, we can more effectively tackle hearing loss in our aging population, mitigate the risks associated with cognitive decline, and improve their overall quality of life, thereby alleviating the burden on a global scale.

Our decomposition analysis provides clear quantitative evidence that population growth and aging are the primary drivers of the rising burden of hearing loss. From 1990 to 2021, global population growth and aging accounted for more than half of the increase in years lived with disability (YLDs) among older adults, while the impact of changes in epidemiological rates was negligible. This pattern is particularly pronounced in low- to middle-SDI regions, where population growth is the dominant factor. This finding is crucial, as it directly informs our predictions for the future: by 2040, the number of cases and YLDs is expected to continue rising, not a prediction of an increasing epidemic risk, but rather an inevitable result of demographic momentum. This highlights that the future challenge will be expanding access to healthcare to address a larger, aging population, rather than merely preventing the rise in incidence. Notably, in areas within the middle socioeconomic development index (SDI) quintile, the impact of demographic factors is the most pronounced, whereas the influence in high-SDI and low-SDI areas is comparatively minor. In low-SDI areas, limited access to healthcare and a lack of medical services, such as audiological evaluations and hearing aids, may lead to an underestimation of the prevalence of hearing loss, resulting in lower reported rates of hearing impairment (70). It is important to note that the population in certain low-SDI areas is predominantly Black, which may also contribute to a reduced likelihood of hearing loss. This phenomenon has been corroborated by both epidemiological (71, 72) and clinical studies (73). Current hypotheses suggest a possible protective effect of melanin on the stria vascularis (74). In areas with medium SDI, higher rates of hearing loss may be attributed to environmental factors, such as noise pollution from heavy traffic (75). Furthermore, social isolation emerges as a critical factor at the intersection of hearing loss and socio-demographic indicators. Older adults with hearing impairment are at an increased risk of social isolation, which can subsequently lead to mental health issues, including depression and cognitive decline (76). The relationship between social isolation and hearing loss is particularly pronounced among older adults, in whom communication difficulties can hinder social interaction and lead to withdrawal from community activities (77). Those with hearing loss often encounter challenges related to memory, orientation, and language skills, which may further exacerbate feelings of loneliness and dependence (78). In contrast, older adults in developed countries typically exhibit higher rates of social participation. This phenomenon may be attributed to a lower prevalence of hearing loss among older adults, as well as improved access to healthcare resources for its prevention and treatment (79).

Compared to previous studies, our research uses the most up-to-date data, takes a global perspective, and employs a more scientifically rigorous approach to investigate trends in disease burden (5, 11, 12). We are not merely repeating existing studies but rather making significant, high-quality expansions and building on their foundation. By using updated data and more advanced analytical methods, we conducted a thorough analysis of a key sub-population (older adults) and reached more detailed, insightful conclusions that hold significantly greater value for guiding future global strategies to prevent and control hearing loss.

The findings of this study have significant implications for public health policy, clinical practice, and future research. From a practical and policy perspective, our results provide a data foundation for targeted interventions. Firstly, the substantial burden in low SDI regions and the absolute increase in projected cases clearly highlight the urgent need for global health organizations and national governments to prioritize hearing care for the aging population, especially in resource-limited settings. This includes integrating cost-effective hearing screening into primary healthcare for older adults and promoting the development of affordable hearing aid markets. Secondly, the APC analysis indicates that gender differences persist across generations, necessitating public health strategies that are sensitively aware of gender issues. These measures should include strengthening occupational hearing protection programs in male-dominated industries and promoting health through activities that target male help-seeking behavior. Finally, decomposition analysis indicates that population growth and aging are the main driving factors, highlighting that future challenges lie in the capacity and accessibility of health systems. Therefore, policymakers must focus on strengthening health systems to manage the growing number of older individuals with HL, rather than merely predicting changes in risk by age. This requires adopting task-sharing models, providing basic hearing care training to primary healthcare workers, and exploring innovative service delivery methods, such as mobile clinics or community rehabilitation.

We not only documented the burden of HL but also employed advanced analytical frameworks (such as APC and decomposition) to differentiate the complex effects of age, time period, and birth cohort. The finding of a declining cohort effect among recently born infants opens new avenues for etiological exploration, suggesting that improvements in early-life factors or pediatric care may have lasting protective effects on hearing in future generations—an assumption that warrants further longitudinal research. In addition, our study indicates a non-linear relationship between SDI and the burden of hearing loss, challenging the assumption of a socioeconomic gradient and suggesting that specific country-level factors, such as environmental noise regulations or cultural attitudes toward hearing loss, are key moderators that should be incorporated into more detailed models of health disparities. Therefore, future research should prioritize mixed-method approaches to identify the drivers of these specific environmental factors.

However, similar to other Global Burden of Disease (GBD) studies, our research has several limitations. First, the estimation methods employed in the GBD framework face inherent challenges, primarily because they depend on multiple data sources. Inaccuracies in these sources, along with the methodologies used to integrate data from different origins, can introduce bias. The aggregation process may inadequately account for the nuances of diverse populations, resulting in generalized conclusions that may not be applicable to specific contexts (12, 18). Furthermore, the reliance on disability weights in GBD studies, which quantify the severity of different health conditions, can be contentious. These weights are derived from population surveys and may not accurately reflect the experiences of all demographic groups, particularly marginalized populations. Consequently, this may lead to either underestimating or overestimating the burden associated with certain diseases. Additionally, access to data is not uniform across regions, which can create information gaps and biased results, especially in low- and middle-income countries where health data collection systems may be underdeveloped, resulting in underreporting or misclassification of diseases and health outcomes (29, 80). Finally, due to limitations in the database, our data may include only a minimal representation of hearing loss attributable to other causes, such as ototoxic drugs or trauma (81, 82). These limitations underscore the necessity for careful consideration when utilizing GBD data to inform health strategies and resource allocation.

## Data Availability

The original contributions presented in the study are included in the article/Supplementary material, further inquiries can be directed to the corresponding authors.

## Footnotes

1
https://ghdx.healthdata.org/

2
https://surveillance.cancer.gov/joinpoint/
