# Supplementary material for: Global burden of hearing loss in people aged 60 years and older, 1990–2021: results from the global burden of disease study
Source: Front Public Health. 2025 Nov 26;13:1606673. doi: 10.3389/fpubh.2025.1606673 (PMC12689307; doi:10.3389/fpubh.2025.1606673)
Supplement: Supplementary file 1 [file Table_1.docx]

**Supplementary Table 1. The Theoretical Basis and Specific Applications of Various Statistical Analysis Methods**

| ****Analytical Approach**** | ****Primary Purpose**** | ****Key Parameters/Target Outcomes**** | ****Rationale for Application in This Study**** |
| --- | --- | --- | --- |
| **Descriptive Analysis & EAPC** | To describe the basic features of the data and quantify the overall temporal trends. | Number of cases, ASRs Estimated Annual Percentage Change (EAPC) and its 95% CI | To provide a foundational overview of the magnitude and direction (increasing/decreasing) of the burden of hearing loss over time at global, regional, and national levels. |
| **Joinpoint Regression** | To identify points in time where a statistically significant change in the trend occurred. | Annual Percentage Change (APC) for each segment Average Annual Percentage Change (AAPC) for the entire period | To move beyond a single overall trend and detect specific years where the trajectory of hearing loss burden significantly accelerated, decelerated, or reversed, which may correspond to historical events or policy changes. |
| **Age-Period-Cohort (APC) Model** | To disentangle the independent effects of three temporal factors: age, calendar period, and birth cohort. | Age effect: RR by age group Period effect: RR by calendar year Cohort effect: RR by birth year | To understand whether the observed changes in burden are due to the aging population (age effect), factors affecting all ages simultaneously (period effect, e.g., new healthcare policies), or factors specific to generations (cohort effect, e.g., childhood nutrition, historical noise exposure). |
| **Decomposition Analysis** | To quantify the contribution of population growth, population aging, and changes in epidemiological rates to the total change in burden. | Absolute and relative contribution of: Population growth Population aging Epidemiological change | To answer the critical question: "Is the increasing number of people with hearing loss primarily due to there being more older adults (aging), more people overall (growth), or a genuine increase in the risk of hearing loss (epidemiological change)?" |
| **Inequality Analysis (SII & CI)** | To measure socioeconomic inequality in the distribution of health burden across countries. | Slope Index of Inequality (SII): Absolute inequality Concentration Index (CI): Relative inequality | To objectively measure the disparity in hearing loss burden between countries with high and low SDI levels and to track how this equity gap has changed from 1990 to 2021. |
| **Bayesian Age-Period-Cohort (BAPC) Prediction** | To forecast future disease burden based on historical trends in age, period, and cohort effects. | Predicted number of cases and ASRs for prevalence and YLDs up to 2040 | To inform public health planning and resource allocation by providing data-driven forecasts of the future burden of hearing loss among the elderly, which is crucial for proactive policy-making. |
