# Supplementary material for: Global burden of hearing loss in people aged 60 years and older, 1990–2021: results from the global burden of disease study
Source: Front Public Health. 2025 Nov 26;13:1606673. doi: 10.3389/fpubh.2025.1606673 (PMC12689307; doi:10.3389/fpubh.2025.1606673)
Supplement: Supplementary file 7 [file Supplementary_file_1.docx]

**Supplementary File 1. Specific inclusion and exclusion criteria for the data**

**[1]** The selection criteria for our data extraction are as follows:

1. Cause: We selected the cause "Hearing loss".

(2) Age: Age: The population from which we extracted data is over 60 years old.

(3) Metrics: Indicators: We collected data on key burden indicators: number of cases, prevalence, years lived with disability (YLDs), and disability-adjusted life years (DALYs). Age-standardized rates (ASRs) are directly extracted or calculated based on extracted data.

(4) Time: Data for all available years from 1990 to 2021 were included to analyze temporal trends.

(5) Location: Data were extracted at the global level, for all 204 countries and territories, and aggregated according to the Socio-demographic Index (SDI) quintiles (high, high-middle, middle, low-middle, low).

**[2]** The exclusion criteria were inherent to the GBD estimation process itself. The GBD study uses complex statistical models to synthesize data from various sources (e.g., surveys, claims data, literature). Data points that are deemed outliers or of low quality during this modeling process are effectively down-weighted or excluded by the GBD modeling framework. Therefore, our analysis relied on the final, model-based estimates provided by GBD, which represent the best available synthesis of all eligible source data according to the standardized GBD methodology.
